# Amount of < 1Hz deep sleep correlates with melatonin dose in military veterans with PTSD

**DOI:** 10.1016/j.nbscr.2021.100072

**Published:** 2021-07-16

**Authors:** Julie Onton, Lu D. Le

**Affiliations:** a Institute for Neural Computation, University of California San Diego, 9500 Gilman Drive, #0523, La Jolla, CA, 92093, USA; b Veterans Affairs San Diego Healthcare System, ASPIRE Center, 2121 San Diego Avenue, San Diego, CA, 92110, USA

**Keywords:** Lo Deep, Slow oscillations, EEG, Spectral

## Abstract

Military veterans with posttraumatic stress disorder often complain of non-restful sleep, which further exacerbates their symptoms. Our previous study showed a deficit in Lo Deep sleep, or slow oscillations, in the PTSD population compared to healthy control sleepers. Because Lo Deep sleep is likely a stage when the brain eliminates protein debris, it is critical to find the cause and effective therapeutics to reverse Lo Deep deficiency. The current study aims to replicate and extend this finding by examining several physiological and medication factors that may be responsible for the Lo Deep deficiency. We recorded overnight sleep electroencephalogram (EEG) via a 2-channel headband device on 69 veterans in a residential treatment facility. Dried urine samples were collected at 4 time points during one day to measure melatonin, cortisol, norepinephrine and other factors. EEG data were transformed into frequency power and submitted to an automated sleep scoring algorithm. The scoring corresponds to clear spectral patterns in the overnight spectrogram but does not align exactly with traditional visual scoring stages. As expected**,** veterans showed decreased Lo Deep (activity < 1 Hz) and more Hi Deep sleep (1–3 Hz activity) than healthy controls, replicating our previous study. Multiple linear regressions showed that melatonin dose and morning urine melatonin correlated with more Lo Deep sleep. Buspirone dose also correlated with more Lo Deep, but only 6 subjects were taking buspirone. Also replicating the findings from our last study were independent reductions of REM sleep with prazosin and sertraline. Other findings included decreased Lo and increased Hi Deep sleep with higher caffeine dose, and less Hi Deep percentage with higher testosterone. Finally, evening cortisol levels correlated with a higher percentage of Wake after sleep onset. These results confirm Lo Deep deficiency in this PTSD population and suggests melatonin as a possible therapeutic to reverse Lo Deep deficiency. This is a critical first step to establishing a systematic sleep assessment and treatment program in this and potentially other populations to prevent future brain pathology.

## Highlights

• Automatic spectral sleep scoring allows identification of Lo vs Hi Deep sleep.
• Veterans with PTSD express less Lo Deep sleep (<1 Hz) than controls.
• Melatonin dose and urine levels correlated with more Lo Deep sleep percentage.
• Prazosin and sertraline correlated with less REM percentage.
• Higher caffeine intake correlated with less Lo Deep and more Hi Deep sleep.

## Abbreviations:

REM: rapid eye movement
EEG: electroencephalogram
PTSD: posttraumatic stress disorder

## 1 Introduction

It is well known that sleep quantity is important for optimal health, but sleep quality is arguably of higher importance. For example, it is known that benzodiazepines can change sleep architecture such that both slow wave sleep and rapid eye movement (REM) are suppressed in favor or light sleep (Proctor and Bianchi, 2012). In the military posttraumatic stress disorder (PTSD) population, complaints about perceived sleep quality, along with the more obvious problems with sleep initiation and nightmares, are nearly universal (Williams et al., 2015; Mysliwiec et al., 2018). However, while various alterations in sleep architecture have been reported (Kobayashi et al., 2007), there has been no clear consensus to explain the perceived lack of restfulness in military veterans with PTSD (Khazaie et al., 2016).

In our last study, we showed that a population of military veterans in a residential treatment facility showed less of a newly described sleep stage called Lo Deep (Onton et al., 2018). Lo Deep is differentiated from Hi Deep by the dominant frequency range expressed. Hi Deep has dominant electroencephalogram (EEG) power in the 1–3 Hz range while Lo Deep is characterized as large non-oscillatory fluctuations in the <1 Hz range (Onton et al., 2016). The significance of this sleep stage is not entirely known since traditional sleep scoring does not differentiate between these two patterns of activity. However, in the research literature, Lo Deep activity is likely equivalent to slow oscillations, which have gained recent attention largely through the emerging field of auditory stimulation to increase the amplitude of slow oscillations (Ngo et al., 2013; Besedovsky et al., 2017). It is also becoming increasingly clear that activity above and below 1 Hz serve vastly different purposes. For example, accumulation of amyloid-β protein, which can lead to plaques or Alzheimer's disease, was negatively correlated with power below 1 Hz, but was positively correlated with activity above 1 Hz (Winer et al., 2020). Similarly, memory consolidation is enhanced with <1 Hz activity, while activity >1 Hz promotes forgetting (Kim et al., 2019). These remarkable findings underscore the need to re-examine the sleep scoring methods currently in use to better delineate functionally distinct stages of sleep.

This study seeks to confirm and extend our previous findings regarding Lo Deep sleep deficiencies in veterans with PTSD using a spectral sleep scoring method that effectively differentiates between activity above and below 1 Hz.

## 2 Material and methods

### 2.1 Participants

Veterans were recruited from the VA San Diego Healthcare System residential recovery treatment program named the ASPIRE center. Participants were admitted regardless of medication use or sleep apnea diagnosis. No manipulation of medications or the use of continuous positive airway pressure (CPAP) machine. All participants gave informed consent and all procedures adhered to Declaration of Helsinki principles. This study was approved by the Institutional Review Board of the Veteran's Administration, University of San Diego and the Human Research Protection Office of the U.S. Army Medical Research and Development Command.

We enrolled 74 participants, of which only 69 participants (2 females) had at least one night of useable data. The reasons for the reduction in number were: dropping out before recordings, device malfunctions and/or user error. In total, 146 nights were used for all analyses except those including urine measurements. Because some urine samples were not collected correctly and could not be used, diurnal samples (for melatonin, free cortisol, cortisone, norepinephrine, epinephrine) included only 52 subjects and 113 nights. Because urine samples were taken on the day between nights 1 and 2, regression analyses of morning samples only used night 1 data (38 nights/subjects), while evening samples only used night 2 data (36 nights/subjects). Hormone samples (for estradiol, estrone, estriol, pregnanediol, allopregnanolone, dehydroepiandrosterone [DHEA], testosterone, epi-testosterone, 5a-dihydro-testosterone, and androstenedione) included 63 subjects and 134 nights because they only required one valid sample from the day. However, because of fluctuating hormone levels from day to day, hormone measurements hormone regressions only used night 2 data (44 nights/subjects).

Table 1 shows the age and questionnaire statistics from the PTSD participants.

**Table 1:** | Age and questionnaire statistics.

	Mean	Stand. Dev.	Median	Min	Max
*Age*	38.6	7.3	37	25	59
*PCL*	57.3	13.7	58	23	80
*PSQI*	12.9	3.7	12	5	21
*DASS Depression*	24.4	11.2	24.5	4	42
*DASS Anxiety*	20.1	10.6	19.5	2	40
*DASS Stress*	27.8	10.1	28.0	4	42
*ESS*	8.0	5.4	7	0	22
*NSI*	48.8	18.7	49	0	82
*ACE*	3.8	2.9	3	0	10

PCL: PTSD Checklist; PSQI: Pittsburgh Sleep Quality Index; DASS: Depression, Anxiety and Stress Scales; ESS: Epworth Sleepiness Scale; NSI: Neurobehavioral Symptom Inventory; ACE: Adverse Childhood Events; Stand. Dev.: standard deviation; Min: Minimum; Max: Maximum.

Control participants used for the comparison of sleep stage percentages were reported previously in Onton et al. (2016) and Onton et al. (2018). In brief, the original control cohort consisted of 70 medication-free healthy sleepers (39 males, 31 females). From these, all males were used, along with 1 randomly selected female to approximate the percentage of females in the PTSD cohort (3%). The average age of this control cohort was 30.5 ± 7.1 (range 19–48) years. Caffeine use was accepted, though advised to be moderate and not close to bedtime. Alcohol use was not allowed on recording nights.

### 2.2 Equipment

EEG devices used for data collection at the forehead were custom made by Cognionics Inc. (San Diego, CA). Devices collected two channels of forehead EEG at 500 Hz at approximately FP1 and FP2 referenced to right mastoid with no hardware filters. The devices also collected photoplethysmographic (PPG) data to extract heart rate and blood oxygen saturation level (SpO2). The devices collected skin temperature, as well as nearby sound and light levels.

### 2.3 Procedure

Participants were asked to sleep with the headband recording device for 3 consecutive weeknights. Instructions were explained, demonstrated, and written out with photos to reference when applying by themselves. The procedure was to rub the forehead and right mastoid with alcohol or wash with soap, washcloth, and water. Standard electrocardiogram stickers were then snapped into the device for the 2 EEG channels, as well as ground and reference leads. When the forehead was completely dry, they would peel the backings off the stickers and carefully apply the forehead module to the middle-center of the forehead, then slip the headband over the head. The headband could then be tightened to just enough to keep the device from sagging off the forehead, but not more, to avoid headache. Then the mastoid sticker could be applied behind the ear on the bottom aspect of the bone to avoid discomfort while sleeping on that side. The instructions were then to get in bed and, when ready to close their eyes to sleep, slide the device to the on position and ensure that the green function light was on, meaning data was being recorded. In the morning, subjects turned off the EEG device then completed a brief sleep log indicating for that night their time to bed, time to rise and subjective sleep quality on a scale of 1–10.

Participants collected their own urine samples without study staff supervision. Samples were taken by dipping a small sheet of absorbent filter paper into a cup of urine or urinating directly onto the paper so that the paper was saturated up to a designated line. Samples were requested at 4 time points on the day after the first overnight EEG recording. The first collection was upon waking, the second was 2 h later (requesting only 8 ounces of liquid be consumed between), 2 h before bedtime, and just before bedtime. All samples were frozen at -20 °F until all subjects were completed, at which time all samples were mailed in a single package to ZRT laboratories (Beaverton, OR) for analysis. The assay included measurements of the following factors: melatonin, cortisol (total average bound + free and free cortisol at each time point), cortisone, norepinephrine, epinephrine, estradiol, estrone, estriol, pregnanediol, allopregnanolone, DHEA, testosterone, epi-testosterone, 5a-dihydro-testosterone, and androstenedione. All values were expressed in μg/g creatine.

### 2.4 Data processing and visualization

Data were processed in Matlab (Mathworks, Natick, MA, USA) to analyze 3 channels of EEG data: FP1, FP2 and an FP1-FP2 bipolar reference (referred to in this report as forehead-forehead, or FF). The FF channel was preferentially used for all analyses when possible because it tends to have less high frequency noise, as explained in Onton et al. (2016). When one forehead channel was corrupted due to detachment, the other mastoid referenced forehead channel was used instead of FF because in most cases the hypnograms were near identical between leads. All hypnograms used were visually inspected for obvious errors such as REM scored as Wake because of artifactual high frequency noise. None used had any such problems.

**Fig. 1:**
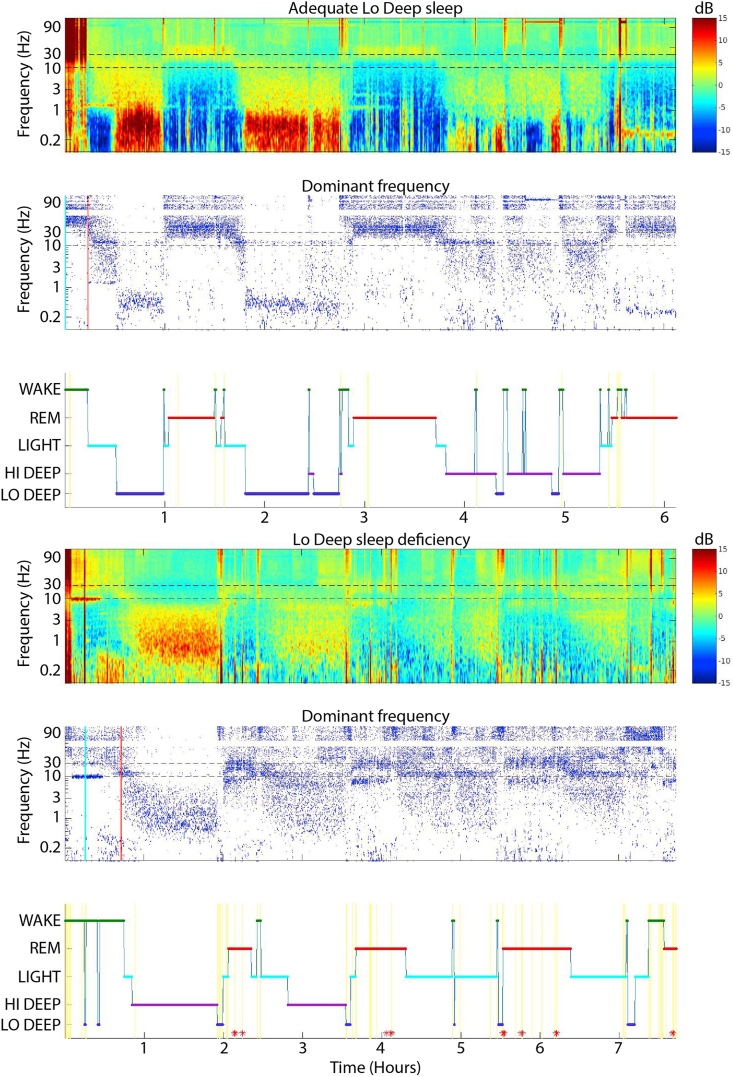
Example sleep reports from two PTSD sleepers, one with adequate Lo Deep sleep (upper report) and one with Lo Deep deficiency (lower report). The top panel of each report is a spectrogram of the overnight power in frequencies between 0.1 and 150 Hz, with the mean log-power across the entire night subtracted from each frequency band. To better visualize the sleep architecture across the night, the middle panel shows a dot at each time point indicating the frequency with the highest relative power. The bottom panel of each report shows the automatically generated hypnogram with sleep stages Wake, REM, Light, Hi Deep and Lo Deep. Vertical yellow lines indicate lower algorithm certainty. Red vertical lines indicate the algorithm's approximation of when sleep began. Cyan vertical lines indicate times of large EEG fluctuations usually consistent with movement. Red asterisks indicate automated switches from an initial Lo Deep designation by the algorithm to REM because REM can sometimes contain low frequency power due to eye movements; lowered spindle power is used to confirm a REM designation in these cases. (For interpretation of the references to colour in this figure legend, the reader is referred to the Web version of this article.)

Details of the EEG data processing are explained in depth in Onton et al. (2016), but in brief, a single channel of EEG data for each night was transformed into frequency power between 0.1 and 150 Hz for every 0.5-s time step, using 3 cycles of Morlet wavelets at the lowest frequency, 30 cycles at the highest and evenly distributed numbers of cycles in between. The average spectrum was calculated from all time points, except those with extreme raw EEG values assumed to be movement and subtracted from the entire night to provide relative power values for each frequency. For visualization on the example sleep reports in Fig. 1, the spectrogram was smoothed with a 40-s moving window to enhance visual detection of spectral patterns. The “dominant frequency” display in the example sleep reports was calculated from unsmoothed relative power values showing a dot, for each time point, at the frequency with the highest power.

SpO2 was estimated from the raw PPG measurements over a 5 s sliding window using the formula presented in Oak and Aroul (2015). Due to device failures, SpO2 was only successfully collected from 59 subjects.

### 2.5 Sleep scoring algorithm

The details of the sleep scoring algorithm are explained in detail in Onton et al. (2016), but in brief, 5 frequency bands were submitted to a custom-designed hidden Markov model and expectation-maximization algorithm to score each 30-s epoch into one of the following sleep stages: Wake: 37–47 Hz, REM: 16–30 Hz, Light: 10.5–16 Hz, Hi Deep: 1–3 Hz or Lo Deep: 0.1–1 Hz. These stages do not correspond exactly to traditional sleep scoring stages. However, REM and Wake should be the same as their visual scoring counterparts, and Lo Deep is likely always scored as S3 in traditional scoring. Light is probably often scored as S2, but Hi Deep is likely a combination of what would traditionally be scored as S2 or S3. This is due to the differences in prominent features and scoring rules between visual scoring and our automatic spectral scoring.

### 2.6 Multiple linear regression

Multiple linear regression models were constructed to examine the influence of SpO2 below which the subject spent 10% of the night (SpO2-10%), 12 medications used on at least 9 experimental nights, and approximate daily caffeine intake on sleep stage percentages. Twenty-seven medications that were used on fewer than 10 nights were excluded from analysis because inclusion decreased the rank of the data below useable limits. In total, 30 participants took between 1 and 3 of the medications that were not included in the regression analysis. SpO2-10% was successfully recorded from 59 subjects, while device failures corrupted the SpO2 data from the remaining 10 subjects. A separate model was created for each sleep stage for each set of co-variates. The Matlab function *robustfit()* was used to be more tolerant of outliers on the resulting model fit. Inputs were z-scored prior to model fitting to normalize the meaning of the regression coefficient outputs (“b value” in Table 3).

**Table 2:** | Percentage of night spent in each sleep stage.

	Light	Hi Deep	Lo Deep	Hi + Lo Deep	REM	Wake
Controls	24.4 ± 6.5	16.6 ± 8.0	22.1 ± 10.1	38.7 ± 7.6	29.4 ± 6.5	7.9 ± 5.7
PTSD	28.0 ± 9.0*	21.7 ± 8.2**	14.0 ± 9.7**	35.6 ± 10.4	26.8 ± 8.4	10.2 ± 6.8*

Values are means ± standard deviations. * Significantly different (p < 0.04), ** Significantly different (p < 0.0001), from corresponding control group by *t*-test, corrected for multiple comparisons. PTSD, posttraumatic stress disorder; REM, rapid eye movement.

**Table 3:** | Results of multiple linear regressions with all nights.

Factor		Light	Hi Deep	Lo Deep	REM	Wake
SpO2 -10%	Coeff.	0.4 ± 1.0	-0.6 ± 1.0	-2.3 ± 1.0	1.9 ± 0.9	0.6 ± 0.8
	p-value	0.7	0.6	0.03	0.04	0.5
Prazosin	Coeff.	0.2 ± 1.0	1.5 ± 0.9	1.4 ± 1.0	-3.0 ± 0.9	0.1 ± 0.8
Ss = 37	p-value	0.9	0.1	0.1	0.001	0.9
Sertraline	Coeff.	-0.9 ± 1.0	0.8 ± 0.9	0.2 ± 1.0	-1.7 ± 0.9	1.4 ± 0.8
Ss = 19	p-value	0.3	0.4	0.8	0.05	0.07
Buspirone	Coeff.	-1.4 ± 1.0	-0.2 ± 0.9	1.9 ± 1.0	-0.003 ± 0.9	-0.2 ± 0.8
Ss = 6	p-value	0.1	0.8	0.05	1.0	0.8
Duloxetine	Coeff.	-1.6 ± 1.0	0.3 ± 0.9	0.9 ± 1.0	-0.5 ± 0.9	-0.06 ± 0.8
Ss = 12	p-value	0.1	0.7	0.3	0.6	0.9
Melatonin	Coeff.	-1.8 ± 1.0	-1.4 ± 0.9	3.1 ± 0.9	0.3 ± 0.9	-0.5 ± 0.8
Ss = 26	p-value	0.07	0.1	0.001	0.7	0.5
Disulfiram	Coeff.	-0.8 ± 1.0	-0.2 ± 1.0	0.2 ± 1.0	-0.7 ± 0.9	1.3 ± 0.8
Ss = 5	p-value	0.4	0.8	0.8	0.5	0.1
Divalproex	Coeff.	0.07 ± 1.1	-1.6 ± 1.0	1.0 ± 1.1	0.1 ± 1.0	0.4 ± 0.9
Ss = 5	p-value	0.9	0.1	0.4	0.9	0.6
Gabapentin	Coeff.	-1.5 ± 1.1	1.7 ± 1.1	1.5 ± 1.1	-0.3 ± 1.0	-0.8 ± 0.9
Ss = 15	p-value	0.2	0.1	0.2	0.7	0.4
Mirtazapine	Coeff.	-0.6 ± 0.9	0.07 ± 0.9	-0.0 ± 0.9	0.0 ± 0.8	0.7 ± 0.7
Ss = 11	p-value	0.5	0.9	1.0	1.0	0.4
Lisinopril	Coeff.	-1.3 ± 0.9	-0.4 ± 0.8	0.6 ± 0.9	0.5 ± 0.8	-0.5 ± 0.7
Ss = 4	p-value	0.1	0.6	0.5	0.5	0.5
Trazodone	Coeff.	-1.2 ± 1.1	1.6 ± 1.0	-0.9 ± 1.1	0.7 ± 1.0	-0.3 ± 0.9
Ss = 25	p-value	0.3	0.1	0.4	0.4	0.8
Bupropion	Coeff.	1.4 ± 2.6	-0.8 ± 2.4	-1.1 ± 2.6	5.7 ± 2.4	-2.9 ± 2.1
Ss = 9	p-value	0.6	0.7	0.7	0.02	0.2
Caffeine	Coeff.	0.9 ± 0.9	1.8 ± 0.8	-1.8 ± 0.9	-0.4 ± 0.8	-0.03 ± 0.7
Ss = 56;	p-value	0.3	0.03	0.05	0.6	1.0
Outliers		3%	2%	5%	5%	3%

coeff = regression coefficient; rounded to nearest 10th place except to avoid 0.0. Red lettering highlights p ≤ 0.05. Positive coefficient indicates a positive correlation with the sleep stage, and conversely a negative coefficient indicates a negative correlation with the sleep stage.

To avoid collinearity, each set of co-variates was tested for collinearity using Matlab's *collintest()* function. For best accuracy in assessing collinearity, data were scaled by dividing each variable by its standard deviation, but the mean was not removed. Collinearity was assessed by calculating condition numbers, which are the ratio of the first singular value and each subsequent singular value. To find which co-variates show collinearity, a proportion of the variance-decomposition was calculated for each co-variate, which would identify co-variates with high proportions as being collinear. Significant collinearity was set as an overall condition number tolerance larger than 30 with 2 or more factors exceeding 0.5 variance-decomposition proportion tolerance within a single condition index. After all collinearities were removed (see below) all final collinearity tests had both <30 overall condition numbers and no two co-variate proportions exceeding 0.5 tolerance in any condition index. For the morning and evening regressions, cortisone had to be removed due to collinearity with cortisol, and epinephrine had to be removed due to collinearity with norepinephrine. For the hormone regression, estrone and androstenedione had to be removed due to significant collinearities. The medication regression could not be submitted in its entirety to the collinearity test because of missing data in the SpO2-10% measurement, but SpO2-10% was found to be significantly correlated with age (p = 0.002, r = -0.28), so age was not used in this regression. In separate regressions without SpO2 but including all other substance factors, age was not found to significantly affect any sleep stage.

## 3 Results

Table 2 shows the comparison of sleep percentages from PTSD and control sleepers. PTSD sleepers showed more Hi Deep sleep and less Lo Deep sleep than control sleepers (p < 0.0001 and p < 0.00001, respectively), but the total amount of Hi + Lo Deep sleep was not significantly different from control sleepers (p = 0.09). PTSD sleepers also showed more Light sleep (p = 0.004) and slightly more Wake (p = 0.04). REM percentage did not significantly differ between the groups (p = 0.06).

For illustration, Fig. 1 shows 2 example sleep reports, one from a PTSD sleeper with adequate Lo Deep and one from a PTSD sleeper showing Lo Deep deficiency. The top panels show the overnight spectrogram with low frequencies at the bottom and high frequencies at the top. The sleeper with adequate Lo Deep shows dark red stretches of low frequency activity during the 2 first cycles, whereas the Lo Deep deficient example shows only Hi Deep activity in all cycles (the <1 Hz activity at the beginning is before sleep onset and likely represents eye movements). The middle panel shows the same overnight sleep record but only displays a dot for each time point indicating the frequency with the highest power. This is especially useful for higher frequency activity during REM that is low power and thus less prominent in the spectrogram display. It is evident in these 2 examples that REM activity can either be only around 25 Hz or can bifurcate into ~6–8 Hz and 25 Hz bands.

All subsequent regression analyses include only PTSD subjects (no control subjects) to show what factors affect sleep stage percentages within this population.

Table 3 shows the results of a series of multiple linear regressions examining the influence of SpO2-10%, 12 medications, and approximate daily caffeine intake on sleep stage percentages.

**Fig. 2:**
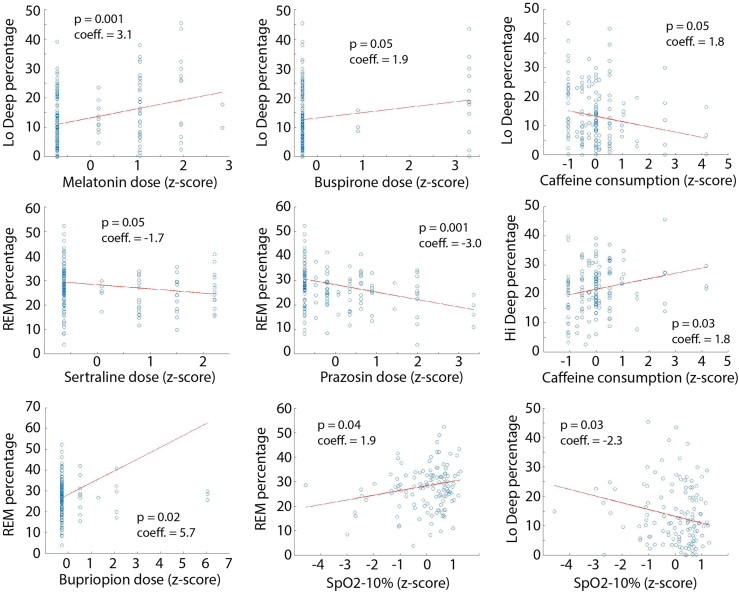
All significant (p ≤ 0.05) correlations from the SpO2 and substance regressions are shown as scatter plots with the red regression lines using the full-factor regression coefficients. (For interpretation of the references to colour in this figure legend, the reader is referred to the Web version of this article.)

Fig. 2 shows the significant correlations found in the multiple regressions shown in Table 3. Substance doses are shown in z-scored values. Each regression line is from coefficients derived from the multi-variate regression model including all factors from Table 3.

Table 4 shows the results of a series of multiple linear regressions examining the influence of urine melatonin, cortisol, norepinephrine and epinephrine on each sleep stage percentage on night 1. Only night 1 was used for these regressions because urine samples were taken on the day after night 1 and morning samples reflect overnight levels.

**Table 4:** | Multiple linear regressions with morning urine factors and previous night.

Morning		Light	Hi Deep	Lo Deep	REM	Wake
Melatonin	Coeff.	-2.5 ± 1.4	-2.0 ± 1.4	4.1 ± 1.4	-2.4 ± 1.4	1.5 ± 1.0
	p-value	0.1	0.2	0.007	0.09	0.1
Cortisol	Coeff.	-.04 ± 1.4	2.3 ± 1.4	-0.04 ± 1.4	-2.4 ± 1.4	1.5 ± 1.0
	p-value	1.0	0.1	1.0	0.09	0.1
Norepinephrine	Coeff.	0.3 ± 1.5	-0.6 ± 1.4	0.06 ± 1.4	1.3 ± 1.4	-1.8 ± 1.0
	p-value	0.9	1.0	1.0	0.3	0.08
Outliers		3%	5%	5%	8%	8%

coeff = regression coefficient; rounded to nearest 10th place except to avoid 0.0. Red lettering highlights p ≤ 0.05. Positive coefficient indicates a positive influence on the sleep stage, and negative coefficient indicates a negative influence on the sleep stage.

Table 5 shows the results of a series of multiple linear regressions examining the influence of urine melatonin, cortisol, norepinephrine and epinephrine on each sleep stage percentage for night 2. Evening urine samples were taken just before bedtime, reflecting levels during the 2 h prior, which could influence subsequent sleep on night 2.

**Table 5:** | Multiple linear regressions with evening urine factors and subsequent night.

Evening		Light	Hi Deep	Lo Deep	REM	Wake
Melatonin	Coeff.	-0.9 ± 1.3	-0.8 ± 1.5	3.7 ± 1.9	-0.1 ± 1.4	-1.6 ± 1.0
	p-value	0.5	0.6	0.06	0.9	0.12
Cortisol	Coeff.	-0.4 ± 1.3	-2.1 ± 1.5	2.2 ± 1.9	-2.2 ± 1.4	3.1 ± 1.0
	p-value	0.8	0.2	0.3	0.1	0.005
Norepinephrine	Coeff.	0.3 ± 1.3	-2.0 ± 1.9	-0.3 ± 1.9	0.3 ± 1.4	1.7 ± 1.0
	p-value	0.8	0.9	0.9	0.8	0.1
Outliers		6%	3%	11%	3%	3%

coeff = regression coefficient; rounded to nearest 10th place except to avoid 0.0. Red lettering highlights p ≤ 0.05. Positive coefficient indicates a positive influence on the sleep stage, and negative coefficient indicates a negative influence on the sleep stage.

Table 6 shows the results of a series of multiple linear regressions examining the relationships between urine estradiol, estriol, pregnanediol, allopregnanolone, DHEA, testosterone, epi-testosterone and 5a-dihydro-testosterone, and each sleep stage percentage for night 2. Hormone levels were assessed by combining all 4 samples from the testing day (2 morning and 2 evening), thus rendering an average hormone level for the day. This hormone state was tested for its influence on subsequent sleep on night 2.

**Table 6:** | Multiple linear regressions with evening hormone factors and subsequent night.

Hormones		Light	Hi Deep	Lo Deep	REM	Wake
Estradiol	Coeff.	1.9 ± 1.7	1.7 ± 2.0	1.2 ± 2.8	-2.8 ± 1.9	-0.9 ± 1.5
	p-value	0.3	0.4	0.7	0.2	0.5
Estriol	Coeff.	-2.8 ± 2.2	1.8 ± 2.5	2.2 ± 3.5	-1.0 ± 2.5	-0.3 ± 1.9
	p-value	0.2	0.5	0.5	0.7	0.9
Pregnanediol	Coeff.	1.1 ± 2.9	-0.9 ± 3.3	-1.7 ± 4.6	0.8 ± 3.2	2.7 ± 2.5
	p-value	0.7	0.8	0.7	0.8	0.3
Allopregnanolone	Coeff.	-1.2 ± 2.9	-3.6 ± 3.0	-.0.5 ± 4.6	4.6 ± 2.9	-1.2 ± 2.2
	p-value	0.7	0.2	0.9	0.1	0.6
Total Cortisol	Coeff.	0.1 ± 1.3	-0.01 ± 1.5	-1.1 ± 2.1	1.0 ± 1.5	0.8 ± 1.1
	p-value	1.0	1.0	0.6	0.5	0.5
DHEA	Coeff.	-1.7 ± 1.5	2.9 ± 1.7	0.4 ± 2.3	-1.4 ± 1.6	0.5 ± 1.3
	p-value	0.2	0.09	0.9	0.4	0.7
Testosterone	Coeff.	2.8 ± 2.2	-5.1 ± 2.5	0.9 ± 3.5	3.0 ± 2.4	0.7 ± 1.9
	p-value	0.2	0.05	0.8	0.2	0.7
Epi-testosterone	Coeff.	-0.9 ± 1.2	0.9 ± 1.4	-0.2 ± 2.0	-2.1 ± 1.4	1.2 ± 1.1
	p-value	0.5	0.5	0.9	0.1	0.3
5a-hydroxy-testosterone	Coeff.	0.9 ± 2.1	3.2 ± 2.4	-1.6 ± 3.3	-1.0 ± 2.3	-0.3 ± 1.8
	p-value	0.7	0.2	0.6	0.7	0.9
Outliers		5%	5%	5%	7%	5%

coeff = regression coefficient; rounded to nearest 10th place except to avoid 0.0. Red lettering highlights p ≤ 0.05. Positive coefficient indicates a positive correlation with the sleep stage, and negative coefficient indicates a negative correlation with the sleep stage.

DHEA: Dehydroepiandrosterone.

**Fig. 3:**
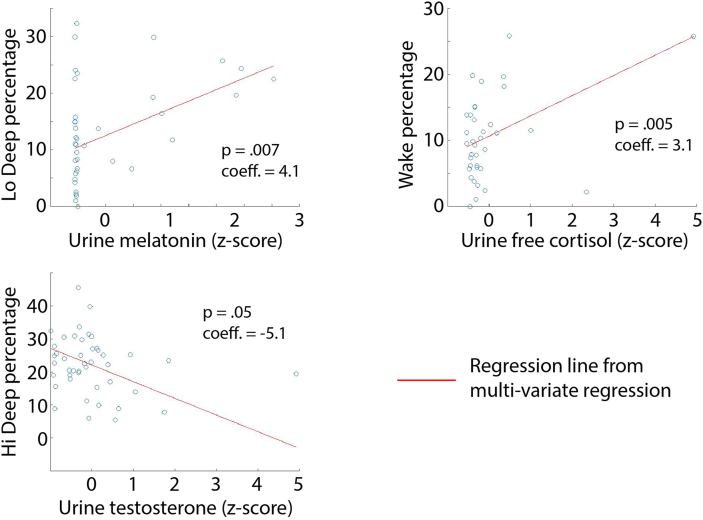
All significant (p ≤ 0.05) correlations from the regressions with urine measures are shown as scatter plots with the red regression lines using the full-factor regression coefficients. (For interpretation of the references to colour in this figure legend, the reader is referred to the Web version of this article.)

Fig. 3 shows the significant correlations in the urine factor regressions. Each regression line is from coefficients derived from the multi-variate regression specific to morning, evening or average sample models. Urine measurements are shown in z-score values as was submitted to the regression analysis.

General subjective sleep quality ratings collected via the PSQI showed that higher Lo Deep percentages correlated with worse subjective overall sleep quality (higher PSQI score indicates worse sleep; p = 0.005; r = 0.23). However, nightly sleep quality ratings did not correlate with the Lo Deep quantity from the same night.

## 4 Discussion

### 4.1 Replication of Lo Deep deficiency

The present study replicates several findings from Onton et al. (2018) showing less Lo Deep and more Hi Deep in a new cohort of subjects drawn from the same residential PTSD treatment facility used in the previous study. Because the current study design did not include a new control group, the present results were compared with the same control group as Onton et al. (2018), with the exception that it was adjusted to a comparable number of female subjects as the current PTSD sample. The control group was younger, on average, than the PTSD populations in both studies. However, in the current study age did not emerge as a significant factor when controlling for sleep apnea and most individual medications used. In the last study, increasing age was associated with less REM, while controlling for gender, experimental group and 4 different medication classes. In any case, Lo Deep percentage was not related to age in either study, suggesting that the lack of Lo Deep sleep in the PTSD population is not merely due to a difference in age between the groups. Also replicating Onton et al. (2018) was a slight increase in Light sleep and Wake after sleep onset in the PTSD populations. These were both marginal compared to the differences in Hi and Lo Deep sleep but were nonetheless replicable and may be true propensities in the sleep patterns of some veterans. The only difference that did not rise to significance in the current study was REM percentage, which was significantly lower in Onton et al. (2018) for PTSD sleepers. However, in the regression analysis with medication doses, prazosin and sertraline were significantly correlated with less REM, suggesting that the trend toward less REM in the PTSD population may be due to medication effects in some individuals. The prazosin correlation with less REM is a replication of our last study, and the sertraline correlation is similar to the previous study wherein several SSRIs grouped together were associated with less REM. Only one other study has quantified the effect of prazosin on REM sleep, but their result showed an increase instead of a decrease in total REM sleep time compared to placebo with a simple REM-tracking device (Taylor et al., 2008). SSRIs are commonly associated with reduced REM sleep quantity (Murdoch and McTavish, 1992), therefore our finding is further corroboration of SSRI influence on REM sleep. Melatonin was not used frequently enough in the previous study to warrant inclusion in the correlation analyses, making the current melatonin results novel and of potential importance for treatment of sleep disorders in the PTSD population.

### 4.2 Lo Deep and slow oscillations

Lo Deep sleep was coined based on the <1 Hz spectral signature of the activity, as distinct from Hi Deep sleep which is between 1 and 3 Hz. The waveforms, however, are distinctly different from each other, Hi Deep demonstrating sinusoidal delta waves and Lo Deep exhibiting large, non-sinusoidal shifts in potential. This distinction has, so far, not entered into standard visual scoring rules, but has been investigated for decades in research literature where the distinct waveforms and brain origins have been deeply investigated (Adamantidis et al., 2019). Slow oscillations, which are the defining feature of Lo Deep sleep, have been recently gaining attention for their role in memory formation (Kim et al., 2019; Kawai et al., 2020) and as a target for non-invasive sleep therapy using sound bursts timed to slow oscillations (Besedovsky et al., 2017). Furthermore, it has been shown that slow oscillations are tied to waves of cerebrospinal fluid washing through the brain (Fultz et al., 2019), which appear increasingly likely to be responsible for clearing detrimental protein build-up that can lead to dementia (Ngo et al., 2013). Thus, a distinct sleep stage to quantify slow oscillations as separate from delta waves in standard sleep scoring should be a priority as sleep medicine moves forward.

### 4.3 Melatonin and Lo Deep sleep

Our finding that melatonin dose and morning urine amount (and a trend toward evening melatonin) were correlated with more Lo Deep suggests a potential therapeutic intervention for Lo Deep sleep deficiency in the military PTSD population. Currently, melatonin is primarily used as a mild sleep-inducing agent (Costello et al., 2014), or for traveling across time zones to reset the internal clock (Lewy et al., 1995). Melatonin has been shown to improve sleep quality in elderly subjects (Garfinkel et al., 1995), who often have low melatonin secretion (Pandi-Perumal et al., 2005) and less deep sleep (Redline et al., 2004). Lo Deep sleep has not been examined in the elderly, but it may be that they also exhibit Lo Deep sleep deficiency.

### 4.4 Other factors associated with sleep architecture

Other factors showed associations with various sleep/wake stages. Of note, the anxiolytic buspirone was associated with increased Lo Deep sleep percentage, similar to melatonin. Buspirone is a 5-HT1A receptor partial agonist that has complex actions in pre- and post-synaptic sites. Serotonin is generally understood to promote wakefulness and to inhibit REM sleep, which is in line with what we and other studies have found for the SSRI sertraline (Winokur et al., 1992; Oberndorfer et al., 2000). Serotonin antagonists increase slow wave sleep (Monti, 2011), which indicates that a complex manipulation of serotonin transmission at 5-HT1A receptors may directly influence Lo Deep sleep production. Alternatively, the anxiolytic properties of buspirone may have a more indirect effect on sleep by allowing for deeper relaxation. In any case, when an anxiolytic is medically indicated buspirone may be a desirable intervention, especially if Lo Deep deficiency is detected.

We found that higher caffeine consumption tended to increase Hi Deep sleep and decrease Lo Deep sleep percentage. Caffeine is a known antagonist at adenosine receptors, which decreases the drowsy feeling induced by adenosine binding to its receptors (Ribeiro and Sebastiao, 2010). Caffeine also has an extraordinarily long half-life, between 2 and 12 h (Blanchard and Sawers, 1983; Benowitz, 1990), such that even morning caffeine can potentially affect sleep at night. A shift toward Hi Deep away from Lo Deep sleep may be one way that caffeine inhibits restful sleep.

By including the overnight SpO2 level below which the subject spent 10% of the night (SpO2-10%), we were able to show that higher SpO2-10% was associated with less Lo Deep and more REM. This makes sense when one considers that our sleep stage measurements are in percentages. Someone who spends a significant amount of time in a low oxygenated state will likely disrupt the initial stages of sleep (i.e., Hi and Lo Deep sleep) and therefore delay transition to REM sleep. In this scenario, that subject would have a higher percentage of deep sleep, but not necessarily a higher absolute amount, because REM would be very minimal and sleep would be split between light and deep sleep, along with awake. That Light and Hi Deep were not significantly affected by SpO2-10% may indicate that Lo Deep is actually more highly represented than Hi or Light, perhaps as a compensatory mechanism for the sleep loss induced by low oxygenation. Thus, our results show that SpO2-10% can significantly skew sleep stage percentages, making SpO2 a crucial measurement to collect along with overnight EEG for improved sleep assessments.

The anti-depressant bupropion was found to positively correlate with REM sleep percentage, which has been reported in the literature previously (Nofzinger et al., 1995). In fact, bupropion has been shown to alter several aspects of REM sleep and these alterations were correlated with the anti-depressant response to bupropion (Ott et al., 2002). Because our study only includes 9 subjects taking bupropion, and the regression line clearly either neglects some data points as outliers and/or adjusts based on other factors, a clear conclusion cannot be drawn from our results alone. However, in combination with previous findings, our results may corroborate an increase in REM sleep with use of bupropion.

In this study, testosterone was associated with decreased Hi Deep percentage. In a study of men over 65, lowered testosterone levels were correlated with less slow wave sleep (Barrett-Connor et al., 2008). Lowered slow wave sleep in gonadectomized mice was reversed when exogenous testosterone was introduced (Paul et al., 2009). Thus, lowered testosterone may affect production of Hi Deep sleep, though there was no correlation with Lo Deep sleep production.

Finally, increased evening cortisol was associated with increased Wake (after sleep onset) percentage. In a study of primary insomnia patients, higher evening cortisol was associated with more wake after sleep onset (Rodenbeck et al., 2002). Both of these findings suggest that chronically dysregulated or phasically released cortisol may degrade sleep quality by promoting wakefulness.

### 4.5 Subjective and sleep stage measures

Given that Lo Deep appears to be an essential component of healthy sleep, and healthy sleep is often encouraged for good mental health, it came as a surprise that the only correlations with self-reported sleep health showed that more Lo Deep actually correlated with worse overall sleep quality ratings. However, it should be noted that ratings for each recorded night did not show any correlation with Lo Deep amount, so a direct connection between subjective sleep quality and Lo Deep sleep percentage cannot be drawn. Furthermore, it is well-known that subjective sleep quality does not correlate well with objective measures, and with deep sleep in particular (Kaplan et al., 2017). Often it is the end of sleep that determines subjective sleep quality. For example, an exhausting nightmare could obscure the replenishing effects of Lo Deep sleep earlier in the night, or an early alarm that leaves the sleeper in a groggy state. While feeling good should be part of good sleep, in a population whose sleep is potentially disturbed in multiple ways, subjective impressions of sleep may not be the best measure of sleep quality.

### 4.6 Spectral sleep scoring

The current study uses a method of sleep scoring that was developed to quickly and reliably recognize macroscopic spectral patterns during sleep. This method produces a hypnogram that closely resembles visual scoring results but will never exactly overlap because of the different features and stage nomenclature used for scoring. These differences are most prominent in the Hi Deep and Light sleep designations, which have no strict equivalent in traditional visual scoring. The main reason Hi Deep is not always scored as S3 is that to be called S3 a 30 s stretch must show clear 0.5–2 Hz oscillations in >20% of the period (Silber et al., 2007). Whereas in the spectral method, the scoring for a 30 s stretch is influenced by the average power of 1–3 Hz oscillations relative to other frequencies and therefore does not need to be present for a particular percentage of the epoch. Similarly, Light sleep does not equate to S2 because Light is simply defined as higher relative spindle frequency power compared to other frequencies. S2, on the other hand, is designated if sleep spindles and K-complexes are longer than 0.5 s and 0.5–2 Hz activity occurs in less than 20% of the epoch (Silber et al., 2007). Lo Deep and REM sleep are likely to align with S3 and REM in the visual scoring nomenclature, respectively. Similarly, Wake, when properly differentiated from REM, should also align with W (visually scored wake). A future direction of this research is to identify the scoring differences between these methods for clinicians and researchers to understand the relationship between the two. Both visual scoring and spectral scoring are valid measures, but the advantages of spectral scoring are the objective consistency and speed with which an algorithm can detect spectral patterns over a whole night of sleep. Once a database of healthy sleeper values is created, this algorithm could be used clinically to identify possible sleep pathologies. The simple spectral display of the actual spectral brain activity for a whole night also provides both a quick way to check the accuracy of the algorithm and to subjectively assess features that are difficult to program into an automated algorithm. Furthermore, spectral scoring only requires a single forehead channel of EEG data, while visual scoring typically requires several scalp channels, eye channels and a chin muscle channel. This makes it very easy to collect large amounts of data for sleep studies that can be compared between subjects and subject groups using the same objective algorithm.

## 5 Conclusion

These results confirm a concerning sleep abnormality that should be further explored in both the PTSD and other populations as it may present a risk factor for later brain disease. Melatonin supplementation should be investigated as a possible treatment for Lo Deep deficiency to help boost Lo Deep sleep with minimal side effects.

## Disclosure

The author reports no conflicts of interest in this work.

## CRediT authorship contribution statement

**Julie Onton:** designed the study, conducted recruitment, informed consent, participant training, Data curation, Formal analysis, data analysis, Writing – original draft, submission. **Lu D. Le:** contributed to study design, recruitment, oversight of study activities, data interpretation, Writing – review & editing.

## Declaration of competing interest

**Julie Onton** and **Lu Le** have no conflict of interests to report for the work presented in this manuscript.
